# Electronic Health Record Transitions—How to Make Them Work

**DOI:** 10.1007/s11606-023-08329-7

**Published:** 2023-10-05

**Authors:** David W. Bates, Raj Ratwani

**Affiliations:** 1 Division of General Internal Medicine and Primary Care, Brigham and Women’s Hospital, Boston, MA USA; 2 Harvard Medical School, Boston, MA USA; 3 Department of Health Policy and Management, Harvard T. H. Chan School of Public Health, Boston, USA; 4 MedStar National Center for Human Factors Engineering in Healthcare, MedStar Health, Washington, DC USA

Nearly all US hospitals have now adopted EHRs, which are complex software products that mediate most of what happens in hospitals today. Not surprisingly, organizations sometimes need to change from one vendor to another, which is remarkably complicated given the many connections involved between applications and areas such as the laboratory and radiology, and the huge array of data which are incorporated. There are many issues involved,^1,2^ but we will focus on how these transitions can be accomplished safely, what can go wrong, and how to minimize the likelihood that problems will occur. Leveraging the science of human factors which is an applied field of engineering to understand user needs and use this information to inform the implementation and use of the new EHR can ease the burdens of EHR transitions.

One of the first issues is selecting a new EHR product. This is typically done by a group selected by the leadership of the hospital or health system. In many instances, if a hospital is part of a group, there are considerable advantages associated with having all the hospitals in the group use the same EHR. The group should consider the main choices and their unique needs and weigh the pros and cons, which include function, usability, interoperability, safety, coverage of key clinical domains, and price, among others.

There are now four inpatient EHR vendors in the USA which had 86%^3^ of the market share as of 2021, so there has been a great deal of consolidation. Any of these EHR vendor products can represent a reasonable choice depending on the size and complexity of the hospital. In the outpatient setting, there are many more vendors, but it makes data exchange dramatically easier if an institution or group of institutions use only one EHR across all settings, although this is still not the norm in most places.

Once an EHR has been chosen, it makes sense to pick a “go-live” date which is generally at least a year out. Over that period, there are innumerable decisions which must be made about configuration. The vendors generally provide a “base” system, and each organization needs to make very large numbers of configuration decisions which affect most of the core systems. Investing time at this stage to truly understand user needs and workflows so that informed configuration decisions can be made will result in a more optimal implementation and reduce the number of changes that will have to be made in the future. Using a human factors approach to understand user needs through interviews, surveys, and observations of clinicians doing their work will enable smarter configuration decisions.

A key issue is what data to bring over from the old electronic health record. The vendors’ preference is generally to bring as little as possible, but that puts a huge burden on providers. The key areas are laboratory tests and radiographic findings (relatively easy), problems (reasonably easy), notes (easy), and medications and allergies (difficult). The latter is a particular challenge because the coding of “Sigs” (how and when to take the medication) is not as well specified as for the other domains, and many errors occur. Still, from the clinical perspective, it is better to bring these data over. However, even when the prescriptions have been imported, it can take a primary care physician 20 or 30 min to redo the active prescriptions for a complex patient. In a setting like the VA, this may take even longer because there may be multiple sources such as different VA systems and non-VA systems and new EHR requirements for entry of data in multiple places.

As go-live approaches, there are typically many issues which are still not resolved, and the temptation to postpone the date may be great. However, this should be avoided at all costs, as organizations need to marshal amounts of resources and it is extremely costly not to go forward. Some vendors have large penalties which are implemented if there are delays, which are appropriate because many temporary workers have to be brought in around go-live.

Generally, go-lives are done on a day which is slow, typically a Saturday. It is critical to have a lot of help on hand to find the large numbers of errors which are identified early on, especially in the first 2–8 weeks, some of which come up often and need to be corrected quickly. A plan should be created to capture and prioritize the EHR issues that need to be addressed. Most vendors and provider organizations use an information technology (IT) ticketing system to track and respond to reported issues. In addition, some issues get reported through patient safety event reporting systems. Having a clear system for capturing these issues, identifying which ones are safety–critical, and prioritizing addressing those issues is important. For example, at one of our institutions, the hospital pharmacy was not in the data dictionary. The sheer number of issues which need to be corrected makes it hard to make all the fixes quickly. Within 2 months, however, performance and patient throughput should be back to normal.

Some of these issues carry serious risks. At one institution, the global default dosing frequency of an important opioid was inadvertently set at a high dosage. While clinicians should know what dose is appropriate for a given drug, in practice they typically assume that what is suggested for an individual is appropriate for that patient. It is thus essential to pick reasonable default dosing frequencies but also to track safety events closely shortly after go-live and rapidly correct those identified. Another more insidious issue which has come up at multiple institutions is that some orders may be placed but not delivered appropriately if interfaces are not working properly. At several institutions, hundreds or more radiography orders have gotten “lost” and were never acted on. This issue can be especially hard to pick up if it only affects some orders as is often the case. However, diagnosing and then deploying appropriate personnel to resolve the issues with substantial risk of harm has to be a top priority.

The next stage is optimization. One of the most important mistakes organizations make often is to fail to develop a strategy and devote enough resources to this stage. There are myriad tiny things which need to be rectified, and it takes a great deal of time to make all the minor changes needed. For one of the authors, the practice location was set incorrectly, and for every patient, this needed to be corrected manually or billing would not occur. This took 9 months to correct. Many changes which affect usability are involved. This is the stage at which users can customize their screens to make them easier to use, but many organizations do not help users with this, and left to their own devices, most users limp along without making changes that could help their performance improve.

There are several strategies organizations can use to optimize their systems. First, organizations should develop ways to understand how their providers are using the EHR by accessing usage data. These data can provide insights on how many clicks are generated or needed to complete an order, and indications of different workflows. While some users may become more efficient after the implementation of an EHR, most do not and need help if they are to improve. Using these data, organizations can identify those providers or teams that need coaching and provide 1:1 sessions for improvement. Second, organizations can look for patterns in these data across providers to identify aspects of the EHR that should be optimized. This may include changing drop-down menus, modifying the location of certain data elements, or changing workflows. Finally, provider surveys and interviews can provide insight into where user needs are not being met so that system improvements can be made to address those needs.

## DO EHR TRANSITIONS CAUSE HARM?

Perhaps the best study on this question asks specifically whether mortality rates increased following an EHR transition.^4^ It found that they do not go up. Another study asked whether bond ratings dropped after an EHR transition and found that they did not.^5^ But close analyses do identify an increase in the number of task switches physicians engage in (a proxy for workload)^6^ and many adverse events that occur after a transition which are almost certainly related to the transition, for example, setting dosages at the wrong level. While it is not possible to eliminate such issues, organizations should strive to minimize their rates and implement safe practices for detecting them rapidly and making corrections. The SAFER guides provide detailed sets of suggestions about how to manage this.^7^

## CONCLUSIONS

Overall, EHR transitions are enormously complicated, as tens of thousands or more decisions need to be made about configuration and inevitably many need adjustments. While these transitions are now a fact of life, as EHRs are being used routinely, managing them effectively is hugely complex, and demands skill, active and intensive management, and significant resources if this is to be done safely and efficiently. The key approaches to doing this safely have emerged, and the process can be handled well, but the degree of complexity is enormous. The VA will be able to make its migration, but it will require close attention, significant resources, and careful management.
